# NaF-PET Imaging for Detection of Early Arterial Microcalcification and Monitoring of Targeted Therapy: A Narrative Review

**DOI:** 10.3390/cells15161496

**Published:** 2026-08-20

**Authors:** Reza Piri, Sepita Taghizadeh, Poul Flemming Høilund-Carlsen

**Affiliations:** 1 Department of Radiology, Herlev and Gentofte University Hospital, 2730 Copenhagen, Denmark; reza.piri.01@regionh.dk; 2 Department of Radiology, Rigshospitalet, 2100 Copenhagen, Denmark; 3 Student Research Committee, Tabriz University of Medical Sciences, Tabriz 516615731, Iran; sepita.taghizadeh18@gmail.com; 4 Department of Nuclear Medicine, Odense University Hospital, 5000 Odense, Denmark; 5 Research Unit of Clinical Physiology and Nuclear Medicine, Department of Clinical Research, University of Southern Denmark, 5230 Odense, Denmark

**Keywords:** ischemic heart disease, microcalcification, sodium fluoride, positron emission tomography

## Abstract

**Highlights:**

- NaF targets hydroxyapatite, the mineral building block of arterial calcification.
- Increased NaF uptake associated with active mineral deposition can be detectable before mineral accumulation becomes apparent as macroscopic calcification on CT.
- NaF-PET appears well suited to demonstrating the efficacy of anti-atherosclerotic drugs and lifestyle interventions.

**Abstract:**

Ischemic heart disease is currently diagnosed mainly through cardiac computed tomography (CT) angiography and functional testing, both of which detect only advanced arterial macrocalcification, at a stage when treatment can merely slow disease progression rather than reverse it. Yet, macrocalcification represents the end product of a much earlier molecular process, which is microcalcification. This process is driven by smooth muscle cell and macrophage apoptosis, matrix vesicle release, and osteogenic phenotypic transitions within the arterial intima, occurring years to decades before mineral deposits become visible on CT. [^18^F]Sodium fluoride (NaF) positron emission tomography (PET) exploits fluoride binding at accessible hydroxyapatite surfaces to detect increased tracer uptake associated with active mineral deposition, including mineralization occurring at a microscopic scale below the direct spatial resolution of clinical PET. Studies demonstrate that anti-atherosclerotic interventions, including statins, and tissue-nonspecific alkaline phosphatase inhibition can suppress NaF uptake even when CT-based calcium scores remain unchanged or continue to rise, a dissociation now also observed in human trials of statins and PCSK9 inhibitors. This review traces the cellular and histological basis of arterial calcification, outlines the principles and limitations of NaF-PET imaging, and evaluates its emerging role—supported by artificial intelligence-based quantification—as a tool for monitoring targeted anti-atherosclerotic treatment.

## 1. Introduction

Cardiovascular disease remains the leading cause of morbidity and mortality worldwide, accounting for an estimated 19.8 million deaths in 2022 alone, equating to 396 million years of life lost and 44.9 million years lived with disability [1]. Atherosclerosis, the pathological substrate underlying most ischemic heart disease and stroke, progresses through a prolonged, largely silent process in which lipid-laden plaques accumulate within the arterial wall over decades before producing clinical symptoms [2]. Rudolf Virchow first proposed inflammation as the initiating event in atherogenesis, and this concept remains foundational: circulating monocytes are recruited to sites of endothelial injury, differentiate into macrophages, and internalize oxidized low-density lipoprotein (LDL) to become lipid-laden foam cells that form the core of the nascent atheroma [3].

Embedded within this inflammatory cascade is a less appreciated but clinically consequential process, which is calcification. Coronary artery calcification (CAC) has long served as a surrogate marker of atherosclerotic burden and is quantified clinically using the Agatston score derived from cardiac computed tomography (CT) [4]. However, this metric captures only macroscopic, late-stage mineralization—by the time calcification is visible on CT, the underlying disease process has often been active for years. The molecular precursor to this visible calcification, microcalcification, forms via distinct cellular mechanisms and is invisible to conventional structural imaging [5].

This diagnostic gap has motivated interest in molecular imaging techniques offering an imaging window into biologically active mineral deposition before macrocalcification becomes detectable by CT. Positron emission tomography (PET) imaging using [^18^F]sodium fluoride (NaF) exploits the tracer’s affinity for hydroxyapatite, the mineral phase of arterial calcification, providing a PET signal associated with active hydroxyapatite deposition at a stage when disease-modifying therapy may still alter its trajectory [6,7,8]. This narrative review integrates current knowledge of the cellular and molecular mechanisms of vascular calcification with the biological basis of NaF-PET imaging and critically examines its emerging role in assessing vascular disease activity and therapeutic response.

Several recent reviews have examined NaF-PET in atherosclerosis from complementary perspectives, including assessment of disease burden and progression, methodological and reproducibility considerations, plaque activity, and technological and translational developments [9,10,11,12]. The present review is intended to provide a distinct, integrated perspective by linking the cellular and molecular mechanisms of vascular mineralization with the biological basis of NaF uptake and its potential application for monitoring therapeutic interventions. Particular emphasis is placed on how processes occurring at the cellular and extracellular level—including phenotypic transition, extracellular vesicle-mediated mineral nucleation, and hydroxyapatite formation—ultimately generate the molecular imaging signal detected by NaF-PET. This mechanistic framework is subsequently used to critically examine the emerging evidence for NaF-PET as an imaging biomarker of therapeutic response.

## 2. Literature Search and Review Approach

This article was designed as a critical narrative review rather than a systematic review. To inform the review, literature searches were conducted in PubMed/MEDLINE, Embase, and Scopus from January 2000 through 1 July 2026. Search concepts included combinations of ‘NaF’, ‘PET’, ‘atherosclerosis’, ‘vascular calcification’, ‘coronary’, ‘carotid’, ‘microcalcification’, ‘treatment’, ‘therapy’ and ‘intervention’. Reference lists of relevant primary studies and recent reviews were additionally examined.

Peer-reviewed human studies were prioritized, particularly prospective intervention studies, randomized controlled trials, longitudinal studies, test–retest investigations, histological validation studies, and studies addressing quantification or technical reproducibility. Mechanistically informative preclinical studies were included where they clarified the cellular basis of mineralization or provided proof-of-concept intervention data. Case reports and very small exploratory studies were retained only where they addressed interventions for which higher-level evidence was limited and are explicitly identified as such. Conference abstracts were excluded.

Because this was a narrative review, study identification and selection were not intended to constitute an exhaustive systematic review, and no meta-analysis or formal validated risk-of-bias instrument was applied. Instead, intervention findings were critically interpreted in relation to study design, sample size, comparator, duration of follow-up, imaging endpoint, statistical uncertainty, and potential sources of bias.

## 3. The Molecular and Histological Basis of Arterial Calcification

### 3.1. Cellular and Molecular Mechanisms of Intimal Microcalcification

Arterial calcification is not a single, uniform process. Depending on its anatomical location within the vessel wall, calcification is classified into two categories with distinct etiologies and clinical implications: Mönckeberg medial calcification and intimal atherosclerotic calcification [13]. Mönckeberg calcification predominantly affects the tunica media of peripheral arteries in patients with long-standing diabetes, chronic kidney disease, hypercalcemia, elevated phosphate, or hyperparathyroidism. It arises largely from osteoblast-like differentiation of vascular cells driven by altered intracellular signaling and calcium-sensing receptor activity, and notably does not typically involve lipid deposition or substantial inflammatory infiltration in its advanced stages [14].

Intimal atherosclerotic calcification, by contrast, is intrinsically linked to atherosclerosis progression and is the process most relevant to ischemic heart disease [15]. It is driven by phenotypic transition of vascular smooth muscle cells toward osteogenic and chondrocyte-like states, together with an active inflammatory cascade involving macrophage infiltration and cytokine release [16]. Because CAC scoring reflects primarily this intimal process [15,17], the remainder of this review focuses on intimal calcification as the substrate for NaF-PET imaging.

### 3.2. Distinct Calcification Phenotypes

Several non-mutually exclusive mechanisms contribute to the initiation of intimal microcalcification. Death of inflammatory cells within the atheroma releases apoptotic bodies that nucleate hydroxyapatite crystal formation [18]; death of smooth muscle cells (SMCs) and macrophages liberates matrix vesicles that serve a similar nucleating function [19]; and phenotypic modulation of SMCs into chondrocyte-like cells promotes bone-like matrix deposition [20,21]. These processes are further exacerbated by reduced expression of endogenous mineralization inhibitors such as osteopontin, fetuin, and pyrophosphate [22,23,24].

Matrix vesicles, extracellular vesicles ranging from exosomes (10–100 nm) to microparticles (100–500 nm) and apoptotic bodies (500–1000 nm), are now understood to play a central nucleating role [25]. Localized collagen degradation allows larger vesicles (100–300 nm) to accumulate within the extracellular matrix, followed by annexin A1-mediated vesicle aggregation [26]. Within these vesicles, phosphoethanolamine/phosphocholine phosphatase 1 (PHOSPHO1) hydrolyzes phosphoethanolamine and phosphocholine, contributing to the generation of inorganic phosphate required for mineral formation from two membrane phospholipids—phosphoethanolamine and phosphocholine—while tissue-nonspecific alkaline phosphatase (TNAP) and nucleotide pyrophosphatases (NPP1, NPP3) liberate additional phosphate from ATP, ADP, and pyrophosphate in the extracellular space [27,28,29]. Pit1 and Pit2 transporters import phosphate into the vesicles while annexins A2, A5, and A6 mediate calcium transport, culminating in nucleation of calcium phosphate, vesicle membrane fusion, and eventual mineral maturation into microcalcification [30,31].

Experimental work has further implicated macrophage-derived matrix vesicles specifically. Under calcium and phosphate concentrations resembling those seen in chronic kidney disease patients on dialysis, macrophage-derived vesicles exhibit heightened calcification potential, mediated by a phosphatidylserine-annexin A5-S100A9 membrane complex that functions as a nucleation site for hydroxyapatite [32]. Given the relative abundance of macrophages over SMCs in rupture-prone plaques, this mechanism may be particularly relevant to the initiation of microcalcification in unstable lesions [33]. A second, independent mechanism involves phenotypic transition of SMCs into osteoblast-like cells that subsequently synthesize osteogenic factors capable of promoting vascular calcium deposition, while a third implicates direct hydroxyapatite crystal formation during apoptosis of SMCs and macrophages themselves [34]. These mechanisms are not mutually exclusive, and current evidence has not established which predominates at any given stage of plaque development.

Taken together, these processes form an interconnected pathway in which cell death and phenotypic modulation promote extracellular vesicle release, followed by local accumulation of calcium and phosphate and nucleation of hydroxyapatite [6]. The newly formed hydroxyapatite surfaces subsequently provide binding sites for NaF, thereby linking the cellular mechanisms of vascular mineralization to the signal detected by NaF-PET.

### 3.3. Histological Progression from Microcalcification to Macrocalcification

The earliest histologically identifiable form of CAC is microcalcification, ranging in size from 0.5 to 15.0 μm, best visualized using von Kossa or Alizarin red staining within regions of pathological intimal thickening [35,36]. These deposits originate predominantly from SMC apoptosis, producing fine microcalcifications, while apoptotic macrophages generate relatively larger punctate deposits, typically located near the internal elastic lamina at the outer margins of the necrotic core [25]. Initial calcification occurring within matrix vesicles measuring only 100 to 700 nm can be resolved only by electron microscopy, underscoring how far this process precedes any form of clinical detectability [35].

Over time, microcalcifications coalesce into larger speckles and fragments, progressing outward from the necrotic core into the surrounding collagenous matrix, eventually forming calcified sheets or plates spanning more than one quadrant of the vessel wall [37]. These sheets may fracture into nodular calcifications that protrude into the lumen, disrupt the endothelial lining, and precipitate acute luminal thrombosis, a mechanism implicated in 2–7% of coronary and 4–14% of carotid thrombotic events identified in pathological studies [38,39].

**Figure 1:**
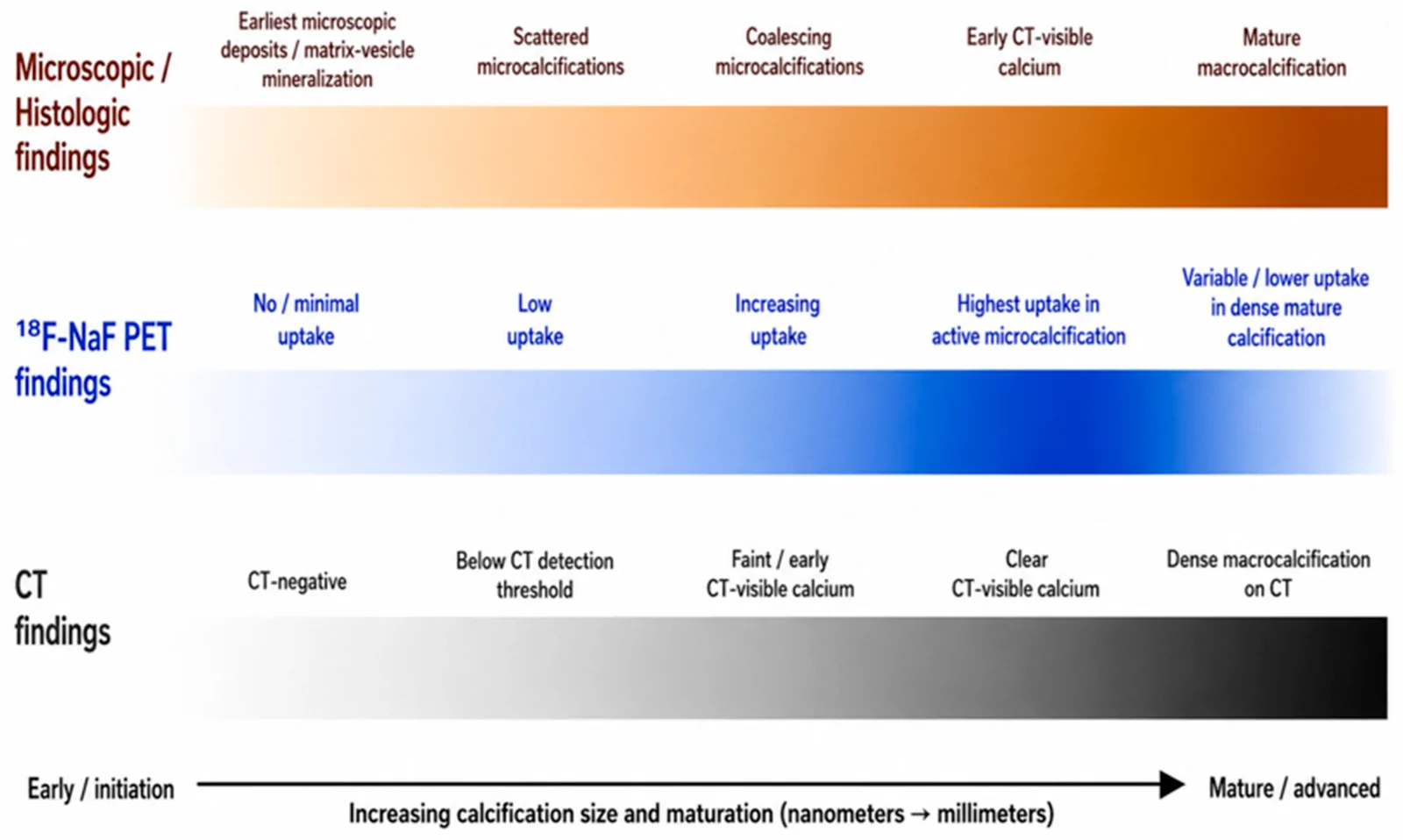
Schematic spectrum of arterial calcification progression and its relationship to microscopic, NaF-PET, and CT findings. Calcification progresses from nanoscale matrix-vesicle mineralization and microscopic deposits toward larger, coalescing deposits and ultimately CT-visible macrocalcification. NaF-PET reflects tracer uptake associated with active hydroxyapatite deposition rather than spatial resolution of individual microcalcifications, with uptake generally increasing during active mineralization and potentially becoming more variable as calcification matures.

This progression mirrors, in several respects, the process of skeletal bone formation, with bone-related proteins such as bone morphogenetic proteins (BMP-1, BMP-4), bone sialoprotein, osteocalcin, osteonectin, osteopontin, and osteoprotegerin identified within calcified arterial tissue [40,41]. Osteoprotegerin, osteopontin, and matrix Gla protein appear at sites of microcalcification even in early plaques—uncarboxylated matrix Gla protein specifically emerges with the transition from adaptive to pathologic intimal thickening—while BMP-2, BMP-4, osteopontin, and osteonectin become more prominent in advanced fibrocalcific lesions [41,42]. Despite this parallel with osteogenesis, true bone formation within the human coronary vessel wall remains rare, and the precise triggers governing the transition from microcalcification to macroscopic sheet calcification remain incompletely understood. Calcification extent also tracks with plaque type and degree of luminal narrowing: it is minimal in early adaptive or pathological intimal thickening, increases progressively in fibroatheroma and thin-cap fibroatheroma, and becomes disproportionately extensive relative to necrotic core size in healed ruptures and fibrocalcific plaques, where diffuse calcification on radiography corresponds histologically to sheet calcification [43]. The progressive relationship between calcification size and its detectability by microscopy, NaF-PET, and CT is schematically illustrated in Figure 1.

### 3.4. Biomechanical Consequences: Microcalcification and Plaque Instability

The clinical significance of microcalcification extends beyond its role as a histological marker—it directly influences plaque biomechanics and rupture risk. Macrocalcification tends to stabilize plaques by acting as a mechanical barrier, whereas microcalcification introduces biomechanical heterogeneity that increases vulnerability [44]. This effect is strongly size-dependent: microcalcifications smaller than 5 μm are predicted to be biomechanically inert, since their associated voids do not undergo explosive growth under tensile stress owing to high surface energy. However, deposits between 5 and 65 μm can amplify plaque tensile stress by up to fivefold, and the interfacial debonding theory proposed by Maldonado and colleagues identifies microcalcifications exceeding 65 μm as a distinct failure mode in which fibrous tissue separates from the mineral surface under threshold stress, precipitating rupture [44]. Consistent with this, nearly all microcalcifications identified within ruptured fibrous caps measured under 65 μm, with a mean diameter of 28 ± 13 μm [36].

Morphology and spatial clustering further modulate risk. Elongated, prolate spheroidal microcalcifications generate substantially higher stress at their poles than spherical deposits, with elliptical microcalcifications of aspect ratio greater than 2 producing up to a fourfold elevation in localized stress concentration compared with a twofold increase around spherical deposits [45,46,47]. Collagen appears to act as a structural scaffold for microcalcification formation, with collagen fiber alignment directly shaping the resulting mineral geometry by guiding matrix vesicle aggregation into elongated forms, while collagen degradation itself accompanies microcalcification development [46,48].

Proximity between adjacent microcalcifications compounds this danger. When the gap-to-diameter ratio (h/D) falls below 0.1, peak tissue stress can increase up to sixfold, and stress concentration rises exponentially as h/D drops below 0.4. Analysis of nearly 35,000 microcalcifications across 22 unruptured plaques found that closely juxtaposed pairs (h/D < 0.4) were rare, occurring in only 3 instances, suggesting either a genuinely low incidence or that such configurations are disproportionately associated with rupture and therefore rarely captured in surviving, unruptured tissue [44]. Laboratory models using silicone-embedded microbeads confirm that closely spaced microcalcifications reduce the ultimate tensile strength (UTS) of fibrous cap tissue by 10.9%, 22.3%, and 39.4% as h/D ratios decreased from 0.86 to 0.60 to 0.54, respectively, with thinner caps showing more pronounced weakening. High concentrations of small hydroxyapatite particles (≤5 μm) similarly reduce UTS and tissue stiffness, an effect attributed partly to a reduction in collagen fiber matrix content [49,50]. Collectively, this body of biomechanical evidence establishes microcalcification—not macrocalcification—as the histological feature most directly implicated in plaque rupture, providing the rationale for imaging techniques capable of detecting it before it accumulates into stabilizing macrocalcific sheets.

## 4. Principles of NaF-PET Imaging

### 4.1. Molecular Basis and Dual-Tracer Complementarity

PET imaging enables visualization of biologically active processes that remain invisible to structural modalities such as CT, ultrasound, or MRI. Two radiotracers dominate atherosclerosis imaging: [^18^F]fluorodeoxyglucose (FDG), which reflects macrophage glucose metabolism and thus arterial inflammation, and NaF, which binds hydroxyapatite and thus reflects microcalcification [51]. Because inflammation and microcalcification are distinct, only partially interconnected biological processes, the two tracers are not interchangeable and often diverge within the same lesion or vascular bed [51].

This divergence has been directly demonstrated in preclinical models. In ApoE-/- mice fed an atherogenic diet, FDG uptake appeared in the aortic arch while NaF uptake instead localized to pulmonary arteries; FDG uptake in the aortic arch was linked to hypoxia rather than inflammation, and FDG did not appear earlier than NaF, challenging the assumption that inflammation strictly precedes calcification [51]. In patients with pseudoxanthoma elasticum, aortic NaF uptake correlated with pulse wave velocity in a pattern opposite to arterial FDG uptake, and the two tracers did not correlate with one another in any arterial segment examined [52]. A dual-tracer PET/CT study of atherosclerotic plaque similarly found that FDG-based inflammation and NaF-based mineral deposition were not directly correlated within the same plaque segments, with mineralization patterns suggesting that active calcification represents a distinct, later biological stage relative to inflammatory activity [53].

**Figure 2:**
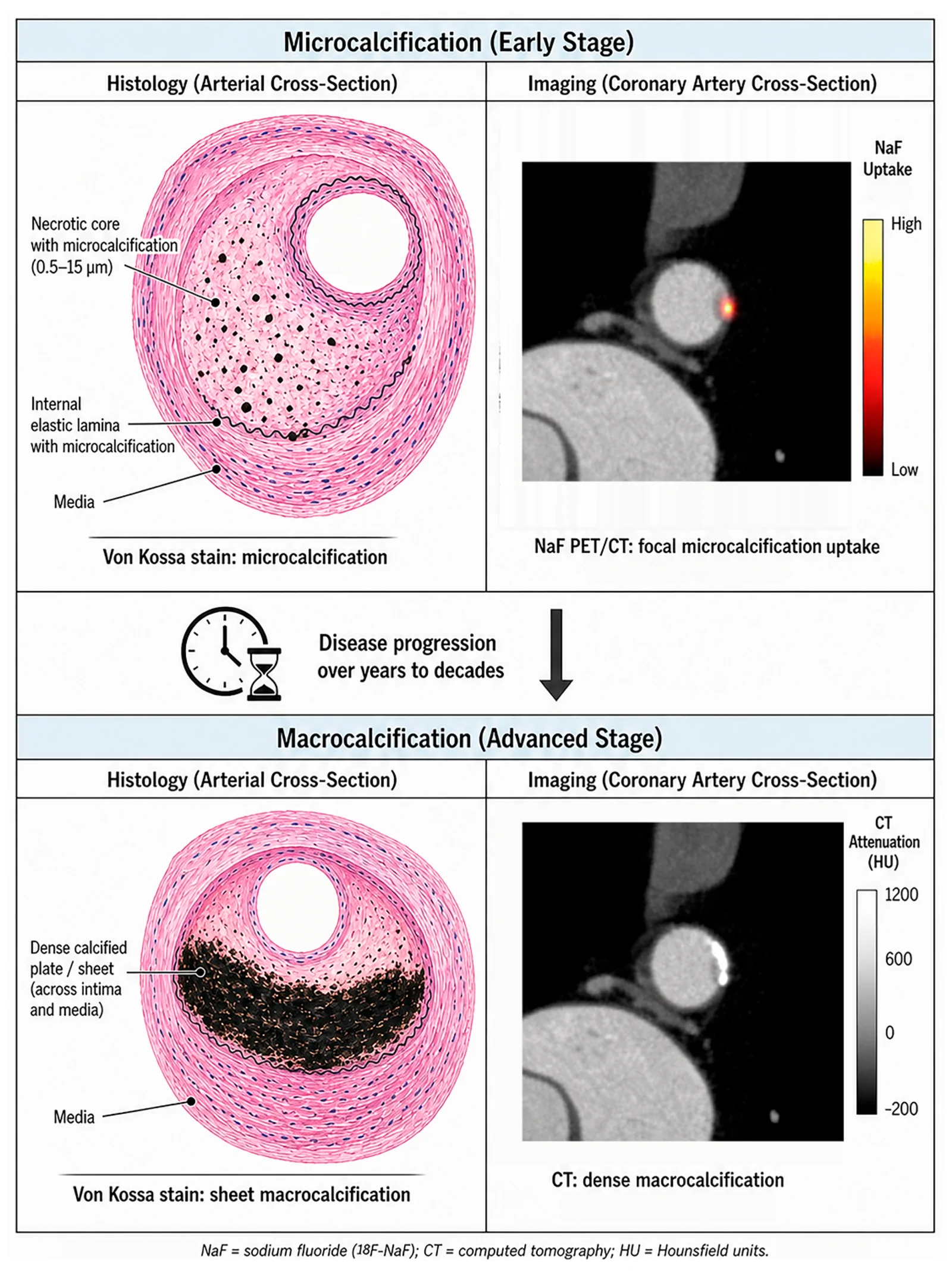
Histology-to-imaging correlation of arterial calcification across disease stages. Top row: Early-stage microcalcification. Left, von Kossa staining of an arterial cross-section with pathological intimal thickening demonstrates small, scattered, dark punctate deposits (0.5–15.0 μm) localized near the internal elastic lamina and at the outer margins of the necrotic core, representing the earliest histologically identifiable calcific deposits arising from smooth muscle cell and macrophage apoptosis. Right, schematic NaF-PET/CT fusion image shows a focal, elevated tracer uptake in a region of the coronary vessel wall without a corresponding dense signal on the unenhanced CT component, illustrating NaF-PET’s ability to detect molecularly active microcalcification before it becomes visible as macroscopic mineral deposition. The PET signal should not be interpreted as spatial resolution of individual histological microcalcifications; rather, it represents tracer accumulation from mineral surfaces within a PET voxel. Bottom row: Advanced-stage macrocalcification. Left, von Kossa staining demonstrates a large, confluent, dense calcified sheet spanning the arterial wall, reflecting coalescence of microcalcific deposits over years to decades. Right, schematic CT image shows a well-defined, dense, hyperattenuating (bright white) calcified plaque within the coronary vessel wall, consistent with a positive Agatston calcium score. Central arrow indicates disease progression from molecular microcalcification to structural macrocalcification over a timescale of years to decades, during which NaF-PET, but not CT, can detect ongoing disease activity. All panels are conceptual illustrations and do not represent matched patient or histological data.

Beyond their distinct biological targets, NaF offers several practical imaging advantages over FDG for vascular quantification: reduced spillover from adjacent avid tissues—a particular problem for FDG given high myocardial glucose uptake—and faster blood clearance, which lowers background blood-pool activity and improves the accuracy of arterial wall uptake measurement [54]. NaF uptake also appears more temporally stable than FDG uptake, which can fluctuate over short intervals in ways that complicate serial monitoring for treatment response [55,56]. Together, these properties position NaF as the more tractable tracer for longitudinal, quantitative studies of calcification biology, while FDG remains complementary for characterizing the inflammatory component of plaque activity. Figure 2 provides a conceptual schematic illustration of the relationship between histological stages of arterial calcification and their expected imaging appearances on NaF-PET/CT and CT. The illustrated panels are intended for conceptual comparison and do not represent experimentally paired histological and clinical images.

### 4.2. Clinical Evidence in Humans

Patients with angina pectoris show significantly elevated pulmonary artery NaF uptake compared with healthy controls [57], and patients with rheumatoid arthritis—a condition associated with accelerated atherosclerosis—demonstrate elevated abdominal aortic NaF uptake alongside higher CT calcium scores, while FDG uptake shows no such difference [58]. In patients with peripheral arterial disease, NaF-PET revealed 30% higher total atherosclerotic burden in non-lower-extremity arteries compared with disease-free controls, driven by elevated carotid and thoracic aortic uptake [59]. In patients imaged for prostate cancer or chest pain, coronary NaF uptake was significantly higher in those with ischemic versus normal myocardial perfusion, and both obstructive and non-obstructive coronary lesions showed higher NaF uptake than disease-free segments, although the correlation with CT calcification itself was often poor—reinforcing that NaF uptake and structural calcium burden are related but non-identical measures [60].

NaF uptake also correlates with plaque vulnerability. Culprit plaques in patients with ST-elevation myocardial infarction show 23–27% higher coronary NaF uptake than plaques in stable angina patients [61]. Intracoronary optical coherence tomography studies have shown that NaF-positive coronary segments have higher lipid arc, greater macrophage prevalence, thinner plaque free wall, and greater total and calcified plaque burden than NaF-negative segments [62]. Similarly, carotid culprit lesions show higher NaF uptake than non-culprit plaques, and thin-cap fibroatheromas—the histological hallmark of vulnerable plaque—cluster within coronary segments exhibiting elevated NaF target-to-background ratios above 1.28 [63]. In carotid stenosis patients, NaF uptake was significantly associated with MRI-defined culprit lesion features including necrosis, intraplaque hemorrhage, ulceration, and calcification [64]. These findings collectively position NaF PET not merely as a marker of calcification burden, but as a potential indicator of plaque instability itself.

An important consideration in vascular NaF-PET is the methodological variability across studies. Differences in tracer activity, uptake time, scanner and reconstruction protocols, motion correction, partial-volume effects, blood-pool correction, region of interest definition, and quantitative metrics such as standardized uptake value, tissue-to-background ratio, and global or focal measures may influence reproducibility and limit direct comparison between studies. These issues are particularly relevant in small and mobile structures such as the coronary arteries [65].

Despite these promising findings, vascular NaF-PET remains primarily a research technique. Its broader clinical implementation is limited by radiation exposure, cost and availability, the need for repeated PET/CT examinations in longitudinal assessment, and the absence of established thresholds for patient selection or interpretation. Moreover, its incremental value over established approaches such as CAC scoring, coronary CT angiography, conventional cardiovascular risk models, and other molecular imaging strategies has not yet been conclusively demonstrated. Importantly, no prospective outcome studies have established that NaF-PET-guided management improves clinical outcomes.

## 5. NaF-PET for Monitoring Targeted Anti-Atherosclerotic Therapy

However, the available evidence spans substantially different levels of evidence, ranging from preclinical studies and individual case reports to observational cohorts and randomized controlled trials. Accordingly, findings should be interpreted in the context of study design, sample size, presence of a comparator, and duration of follow-up. The most clinically compelling application of NaF-PET lies in its apparent ability to detect treatment response at a molecular level, even when structural imaging shows no change—or paradoxically, an increase—in calcification.

### 5.1. Pharmacological Interventions in Preclinical Models

Pharmacological interventions show similarly dissociated effects between molecular and structural imaging. In a rabbit model combining cholesterol-enriched diet with balloon injury of the abdominal aorta, animals treated with atorvastatin (5 mg/kg/day) showed only a 2% increase in NaF uptake from baseline over 18 weeks, compared with a 20% increase in untreated controls—despite total calcium density, measured by von Kossa staining, increasing by 235% in the atorvastatin group relative to untreated animals. Notably, although overall calcium density rose sharply with statin treatment, the areas of microcalcification within plaque were significantly lower, and microcalcification deposits co-localized more closely with regions of elevated NaF uptake, suggesting a shift toward more mature, stable macrocalcification alongside suppression of the biologically active microcalcific component [66].

Vitamin K status has also been examined mechanistically. In ApoE-/- mice maintained on a Western-type diet for twelve weeks followed by a further twelve weeks on control, advanced Western, vitamin K, or Warfarin diets, the Warfarin group—which impairs vitamin K-dependent calcification inhibitors—developed spotty aortic calcifications on CT corresponding to dense mineralization on Alizarin Red staining, and showed the highest NaF uptake in the aortic arch and left ventricle alongside the advanced-diet group. The vitamin K supplemented group had the lowest NaF uptake after controls, while the advanced Western-diet group showed elevated NaF uptake without developing CT-visible spotty calcification at all, directly demonstrating that NaF-PET can detect active microcalcification in the complete absence of structural CT findings [67].

Direct inhibition of TNAP, the enzyme central to matrix vesicle-mediated mineralization, was evaluated using NaF-PET/CT, ex vivo osteosense imaging, and in vitro Alizarin red staining in ApoE-deficient mice. TNAP inhibition prevented aortic calcification both in vivo and in human vascular smooth muscle cells, and additionally reduced blood cholesterol and triglyceride levels, without adversely affecting skeletal bone structure—raising the possibility of a targeted anti-calcific therapy with a favorable off-target metabolic profile [68]. By contrast, a study of semaglutide, a glucagon-like peptide-1 receptor agonist, in a non-diabetic rat model of atherosclerosis found reduced uptake of the macrophage-tracking tracers 64Cu-DOTATATE and FDG, but no change in NaF uptake, supporting the interpretation that semaglutide’s vascular benefit operates through suppression of inflammatory macrophage activity rather than direct inhibition of the calcification pathway [69].

### 5.2. Human Intervention Studies

In a notable case report, a 64-year-old man with mild hypercholesterolemia and a history of limited teenage smoking underwent baseline NaF-PET/CT, which showed a mixed plaque near the right coronary artery ostium, a smaller plaque in the left main coronary artery, and minor left anterior descending artery calcification. After six months of rosuvastatin 10 mg/day, aspirin 75 mg/day, and lifestyle modification, his LDL cholesterol fell by 65%. A repeat scan showed an unchanged CT appearance and unchanged Agatston score, yet revealed a 37% decrease in focal NaF uptake in the right coronary artery plaque and a 40% decrease in the left main plaque, with no new hotspots detected anywhere, including the left anterior descending artery [70]. This case offered an early demonstration that molecular imaging can capture a therapeutic response invisible to structural CT.

This pattern was formally replicated in a larger cohort of 38 patients, most of whom had diabetes and were classified at high or very high cardiovascular risk [71]. In this group, the highest baseline NaF uptake was most commonly observed in the abdominal aorta, followed by the descending aorta, aortic arch, and carotid arteries. Patients with subclinical atherosclerosis and significant plaque NaF uptake were treated with rosuvastatin 20 mg/day for six months, resulting in a statistically significant 19.2% reduction in maximum plaque NaF uptake, with median target-to-background ratio falling from 1.96 to 1.53. Together, the case report and cohort data indicate that statin therapy can measurably suppress microcalcification activity well before any corresponding change becomes apparent on CT. Together, these findings suggest that statin therapy may suppress NaF-defined microcalcification activity; however, the evidence remains limited by the small sample size and non-randomized nature of the available human studies.

The PCSK9 inhibitor evolocumab, a monoclonal antibody that blocks hepatic LDL receptor degradation and thereby enhances LDL clearance, has shown comparable effects in a cohort of 47 patients (mean age 61.8 years, 87% male) with extensive non-calcified coronary plaque and 196 evaluable lesions [72]. Over 18 months of serial coronary CT angiography and NaF PET, evolocumab significantly reduced both coronary microcalcification activity, from a mean of 1.35 to 1.08, and lesion-level NaF target-to-background ratio, from 1.73 to 1.62. The proportion of lesions classified as high microcalcification activity fell from 34.1% to 21.3%, and high NaF uptake lesions fell from 63.8% to 55.6%. Reductions in microcalcification activity occurred in most participants, while changes in target-to-background ratio were more variable, suggesting these two quantitative metrics may capture partially distinct facets of the underlying biological response to intensive lipid-lowering therapy.

Not all interventions have shown benefit. A double-blind, placebo-controlled 2 × 2 factorial trial of vitamin K1 (10 mg/day) and colchicine (0.5 mg/day) in 149 type 2 diabetic patients with existing CT coronary calcification found no significant effect of either agent, alone or combined, on coronary, aortic NaF target-to-background ratio over three months or Agatston score [73]. A subsequent post hoc analysis applying a revised upper limit of normal—defined from a separate zero-calcium diabetic cohort—suggested that vitamin K1 independently decreased the odds of developing new NaF-positive lesions in the coronary arteries (odds ratio 0.35), aorta (odds ratio 0.27), and both territories combined (odds ratio 0.28) [74]. Given its randomized, placebo-controlled design, the primary negative findings of this trial provide stronger evidence than the subsequent post hoc analysis, which should therefore be considered exploratory. This illustrates how threshold definitions can materially alter the interpretation of otherwise negative trial data.

Dietary intervention data add further nuance to this body of evidence. In eleven healthy older adults without clinical cardiovascular disease, six months of supplementation with high-polyphenol extra virgin olive oil significantly reduced both FDG and NaF uptake, indicating attenuation of both arterial inflammation and microcalcification activity [75]. Standard extra virgin olive oil produced no significant effect, while refined olive oil was associated with increased NaF uptake, suggesting active progression of microcalcification. Although these findings suggest a potential effect of polyphenol-rich olive oil on vascular NaF uptake, the very small sample size limits definitive conclusions and the results should be considered hypothesis-generating.

Overall, the human intervention literature remains heterogeneous in study design, sample size, treatment duration, and NaF-PET quantification methods. While several observational studies suggest treatment-related reductions in NaF uptake, randomized evidence remains limited, and larger controlled studies are required to establish the reliability of NaF-PET as a treatment-response biomarker.

### 5.3. Natural History and Prognostic Value

Beyond intervention monitoring, longitudinal studies underscore the slow, variable natural history of arterial NaF uptake, which contrasts with the more consistently progressive nature of CT calcification. In healthy controls followed for two years, global cardiac NaF uptake changed minimally despite a measurable 6% decrease in CT-calcification within the same regions of interest. This finding is reinforced by a dedicated two-year longitudinal study comparing carotid and aortic NaF uptake in 29 healthy controls and 20 angina pectoris patients: aortic partial-volume-corrected mean uptake changed only marginally over two years in both groups (1.14 to 1.29 in angina patients versus 0.99 to 0.95 in healthy controls), with a similar pattern observed in the carotids, and baseline NaF uptake did not predict subsequent change in CT-calcification [76]. Notably, angina patients showed a persistent, though statistically insignificant, trend toward higher NaF uptake than healthy controls at both time points, consistent with a slow but continuously active microcalcification process even under statin therapy, which half of the angina cohort was receiving; NaF uptake across all aortic segments also correlated positively with age [76]. Among patients with pseudoxanthoma elasticum, only two of fourteen showed a two-year increase in CT-calcification of major extracardial arteries, while NaF uptake metrics remained unchanged or even decreased. In a small cohort with 51 CT-defined coronary lesions, already-low NaF uptake showed no change over a follow-up period of three to five years [52].

This slow natural history appears to be modulated by sex and cardiovascular history, according to a separate two-year NaF-PET/CT study in patients with type 2 diabetes. Piri et al. found that microcalcification progression differed significantly by sex, with menopausal women—particularly those without a prior history of cardiovascular events—showing disproportionately higher NaF uptake progression than men or women with established cardiovascular disease [77]. This finding suggests that hormonal status may independently influence microcalcification biology in diabetic patients, adding a further layer of heterogeneity to the already variable natural history of arterial NaF uptake, and raising the possibility that future NaF-PET-based trials may need to stratify or adjust for sex and menopausal status when interpreting longitudinal uptake changes.

Prognostically, baseline NaF positivity predicts future events across several independent cohorts. Among patients with known coronary artery disease, myocardial infarction occurred only in those with abnormal baseline coronary NaF uptake [78]. In diabetic patients, calcification progression was more common in NaF-positive than NaF-negative coronary arteries, and in patients with multivessel disease, more rapid CT-calcification progression—and increase in calcium score—occurred specifically in coronary segments with elevated baseline NaF uptake [79]. In a large analysis of 461 patients with cardiovascular disease followed for approximately one year, progression of thoracic aortic calcium volume correlated with baseline thoracic aortic NaF activity; over six years of follow-up, elevated baseline thoracic aortic NaF uptake was specifically associated with subsequent ischemic stroke, while elevated coronary NaF activity was instead associated with myocardial infarction, suggesting that NaF PET may carry vascular bed-specific prognostic information not captured by a single global calcification score [80]. In one distinct clinical setting, examination of coronary artery bypass grafts found that arterial and venous grafts were entirely devoid of NaF activity nearly three years after implantation, while native, non-bypassed coronary arteries proximal to the graft anastomosis showed three times higher NaF uptake and greater calcium score progression than bypassed segments—an intriguing observation suggesting that surgical revascularization or altered hemodynamics may locally suppress microcalcification activity.

### 5.4. The Emerging Role of Artificial Intelligence

Artificial intelligence has increasingly been investigated specifically for automated analysis of vascular NaF-PET/CT. CNN-based approaches for segmentation of the aorta and common carotid arteries have demonstrated substantially faster analysis compared with manual segmentation while providing broadly comparable quantitative uptake measurements [81,82,83,84]. More recently, an nnU-Net-based model for coronary artery segmentation was evaluated in 141 patients, with 113 used for model training, and demonstrated segmentation performance comparable to intra-observer variability. The same study further evaluated the reproducibility of PET/CT radiomic features derived from manual and AI-based coronary segmentations, illustrating the potential of AI not only for segmentation but also for reproducible quantitative image analysis [84].

Nevertheless, current evidence remains predominantly based on internally validated datasets, and further evaluation across independent populations, scanners, reconstruction protocols, and institutions is required. AI may substantially reduce processing time and observer-related variability, but it cannot overcome fundamental limitations of PET imaging, including limited spatial resolution and partial-volume effects. Consequently, external validation, robustness to scanner and protocol variation, and reproducibility of downstream NaF uptake measurements remain important priorities.

## 6. Conclusions

Arterial calcification begins as a molecular process—governed by matrix vesicle biology, SMC and macrophage apoptosis, and osteogenic phenotypic transitions—long before it becomes visible as macrocalcification on conventional imaging. NaF-PET imaging bridges this gap, offering a window onto microcalcification activity at a stage when the underlying biology remains dynamic and potentially reversible. Accumulating preclinical and clinical evidence demonstrates that anti-atherosclerotic interventions can measurably reduce NaF uptake independent of, or even in the setting of rising, CT-based calcium scores. At the same time, negative and mixed findings—from vitamin K trials to semaglutide—illustrate that not every therapeutic mechanism engages the calcification pathway detectable by NaF, underscoring the tracer’s specificity rather than representing a limitation of the imaging technique itself. As quantification methods mature, particularly through AI-assisted segmentation, NaF-PET represents a promising molecular imaging biomarker of active vascular calcification, with particular potential for treatment-response assessment and use as an imaging endpoint in clinical trials. However, its incremental clinical value over established anatomical and risk-based assessment remains to be determined. At present, the most plausible role of NaF-PET is therefore not routine population screening, but rather the assessment of biologically active vascular calcification in selected high-risk patients and, particularly, as an imaging biomarker in clinical trials evaluating anti-atherosclerotic therapies. Its potential value may be greatest during stages of active mineral deposition before extensive mature macrocalcification predominates. However, cost, limited availability, radiation exposure, and the absence of established clinical thresholds currently restrict broader routine application.

## Data Availability

No new data were created or analyzed in this study. Data sharing is not applicable to this article.

## Abbreviations

The following abbreviations are used in this manuscript:

ADP	adenosine diphosphate;
AI	artificial intelligence;
ApoE-/-	apolipoprotein E knockout;
ATP	adenosine triphosphate;
BMP	bone morphogenetic protein;
CAC	coronary artery calcification;
CNN	convolutional neural network;
CT	computed tomography;
[^18^F]FDG	fluorine-18 fluorodeoxyglucose;
[^18^F]NaF	fluorine-18 sodium fluoride;
[^64^Cu]DOTATATE	copper-64 DOTA-(Tyr^3^)-octreotate;
h/D	gap-to-diameter ratio;
LDL	low-density lipoprotein;
MRI	magnetic resonance imaging;
NPP1/NPP3	nucleotide pyrophosphatase/phosphodiesterases 1 and 3;
PCSK9	proprotein convertase subtilisin/kexin type 9;
PET	positron emission tomography;
PET/CT	positron emission tomography/computed tomography;
PiT1/PiT2	type III sodium-dependent phosphate cotransporters 1 and 2;
SMC	smooth muscle cell;
ST	ST segment;
TNAP	tissue-nonspecific alkaline phosphatase;
UTS	ultimate tensile strength.
